# AI-Derived Pericardial Effusion Volume and Long-Term Mortality After Transcatheter Aortic Valve Implantation

**DOI:** 10.3390/jcm15166388

**Published:** 2026-08-18

**Authors:** Gretha Hecke, Nikolaus Clodi, Bernhard Scharinger, Matthias Hammerer, Laura Preuss, Uta C. Hoppe, Klaus Hergan, Elke Boxhammer, Christoph Knapitsch, Nikolaos Schörghofer

**Affiliations:** 1 Department of Radiology, Paracelsus Medical University, 5020 Salzburg, Austrian.clodi@salk.at (N.C.);; 2 Division of Cardiology, Department of Internal Medicine II, Paracelsus Medical University, 5020 Salzburg, Austria

**Keywords:** transcatheter aortic valve implantation, pericardial effusion, artificial intelligence, computed tomography, hemodynamic congestion

## Abstract

**Background:** Pericardial effusion is a common finding on pre-procedural computed tomography (CT) in patients undergoing transcatheter aortic valve implantation (TAVI). However, its clinical significance remains uncertain. Using artificial intelligence (AI)-based image analysis, we sought to determine whether pericardial effusion represents an incidental imaging finding, a marker of hemodynamic burden, or a prognostically relevant phenotype. **Methods:** This retrospective single-center study included 470 consecutive patients undergoing TAVI for severe aortic stenosis. Pericardial effusion volume was quantified using an AI-based CT segmentation workflow and categorized as no effusion (0 mL), trace effusion (>0–<5 mL), small effusion (5–<30 mL), or larger effusion (≥30 mL). Associations with baseline clinical and echocardiographic characteristics were assessed. Long-term mortality was evaluated using Kaplan–Meier analysis, Cox regression models, and restricted cubic spline analyses. **Results:** Detectable pericardial effusion was present in 276 patients (58.7%), although larger effusions were uncommon (5.1%). Increasing pericardial effusion volume was associated with a higher prevalence of atrial fibrillation (*p* = 0.001), higher systolic pulmonary artery pressure (*p* = 0.004), and lower TAPSE/sPAP ratios (*p* = 0.007). In contrast, left ventricular ejection fraction and transvalvular gradients did not differ across effusion categories. During long-term follow-up, no significant differences in mortality were observed between pericardial effusion groups (log-rank *p* = 0.46). Pericardial effusion volume was not associated with mortality when analyzed as a continuous variable (HR 1.00, 95% CI 0.996–1.01; *p* = 0.842), after logarithmic transformation (adjusted HR 0.91, 95% CI 0.76–1.08; *p* = 0.293), or in restricted cubic spline analyses. **Conclusions:** AI-derived pericardial effusion volume identifies a hemodynamic phenotype characterized by atrial fibrillation, pulmonary hypertension, and impaired right ventricular–pulmonary arterial coupling. Despite these associations, pericardial effusion volume does not independently predict long-term mortality after TAVI, suggesting that it reflects cardiovascular congestion and remodeling rather than a prognostically relevant phenotype.

## 1. Introduction

Not every abnormality detected on pre-procedural CT imaging is clinically meaningful. Some findings are merely incidental, others reflect underlying disease severity, and a few identify distinct phenotypes associated with adverse outcomes. Distinguishing between these possibilities remains one of the central challenges of modern cardiovascular imaging.

Pericardial effusion is frequently encountered in patients undergoing transcatheter aortic valve implantation (TAVI) [1]. While large effusions may warrant immediate clinical attention, smaller fluid collections are often dismissed as incidental findings and rarely influence clinical decision-making [2]. Yet, pericardial fluid accumulation may represent more than a benign bystander. It may reflect elevated cardiac filling pressures, systemic congestion, impaired ventricular interaction, chronic inflammation, or advanced cardiovascular aging [3,4,5,6,7]—processes that are highly prevalent among elderly patients with severe aortic stenosis.

Despite its frequent occurrence, the clinical significance of pericardial effusion in the TAVI population remains poorly defined. Previous studies have primarily focused on procedural complications or relied on echocardiographic assessments using qualitative or semi-quantitative grading systems [1,8,9]. Consequently, it remains uncertain whether pre-existing pericardial effusion represents an incidental imaging finding, a marker of hemodynamic burden, or a distinct prognostic phenotype associated with adverse long-term outcomes.

Recent advances in artificial intelligence (AI) have created new opportunities to extract quantitative information from routinely acquired cardiovascular imaging. Automated image analysis now enables reproducible volumetric assessment of anatomical structures that previously received limited attention in clinical research [10,11]. Applied to the pericardial space, AI-based analysis allows objective quantification of pericardial effusion volume across the entire spectrum of disease, potentially providing a more comprehensive characterization than conventional categorical classifications [12].

The relevance of such an approach may be particularly important in patients undergoing TAVI. Severe aortic stenosis is increasingly recognized as a systemic disease characterized not only by valvular obstruction but also by complex interactions between ventricular remodeling, pulmonary hypertension, atrial dysfunction, congestion, frailty, sarcopenia and biological aging [13,14,15,16]. Within this framework, pericardial effusion may represent a readily visible imaging marker integrating several of these pathophysiological pathways.

Therefore, the aim of the present study was to investigate the association between AI-derived CT-based pericardial effusion volume, baseline clinical and echocardiographic characteristics, and long-term mortality in a large cohort of patients undergoing TAVI.

## 2. Materials and Methods

### 2.1. Study Design and Population

This retrospective single-center observational cohort study was conducted at Paracelsus Medical University Hospital, Salzburg, Austria, and included consecutive patients who underwent TAVI for severe symptomatic aortic stenosis between January 2016 and June 2022.

A total of 585 consecutive patients were initially screened for inclusion. Pre-procedural computed tomography (CT) imaging was unavailable in 46 patients. An additional 69 CT datasets were excluded because image quality was insufficient for reliable automated image analysis and volumetric assessment of the pericardial space. The final study population therefore consisted of 470 patients.

All patients underwent comprehensive pre-procedural evaluation according to institutional standards and contemporary guideline recommendations for TAVI. Clinical, laboratory, echocardiographic, procedural, and follow-up data were obtained from electronic medical records and institutional databases.

The study was conducted in accordance with the principles of the Declaration of Helsinki and was approved by the local ethics committee (EK-Nr. 1082/2024).

### 2.2. CT Acquisition

All patients underwent routine pre-procedural electrocardiography-gated computed tomography angiography (CTA) for TAVI planning. CT imaging extended from the lung apex to the proximal femoral arteries and was performed using either a 256-slice CT scanner (Revolution, General Electric Healthcare, Chicago, IL, USA) or a 128-slice dual-source CT scanner (Somatom Definition AS+, Siemens Healthcare, Erlangen, Germany).

Tube voltage (80–120 kVp) was adapted according to patient size, and automatic tube current modulation was applied. Contrast enhancement was achieved using a bolus-tracking technique with administration of 100 mL of non-ionic iodinated contrast agent followed by 70 mL saline at a flow rate of 3.5–5.0 mL/s.

Images were reconstructed using a soft-tissue kernel with a slice thickness of 3 mm and a reconstruction interval of 2 mm.

### 2.3. AI-Based Pericardial Effusion Quantification

Pericardial effusion volume was quantified using an artificial intelligence (AI)-based image analysis workflow applied to routine pre-procedural CT datasets. DICOM datasets were converted to NIfTI format using dcm2niix (Chris Rorden, University of South Carolina, Columbia, SC, USA). Automated segmentation was subsequently performed using TotalSegmentator (version 2.12.0; University Hospital Basel, Basel, Switzerland), a deep learning-based framework for comprehensive anatomical segmentation of CT imaging [17]. TotalSegmentator was operated in a Python 3.13 environment running on Ubuntu 24.04 via Windows Subsystem for Linux 2 (WSL 2; Microsoft Windows 11). Computations were performed on a dedicated workstation equipped with an NVIDIA GeForce RTX 5090 GPU, an AMD Ryzen 9 9950X3D processor (Advanced Micro Devices, Inc., Santa Clara, CA, USA), and 64 GB DDR5 RAM. No custom preprocessing, resampling, or postprocessing steps were applied beyond the standard procedures implemented within the TotalSegmentator pipeline.

**Figure 1:**
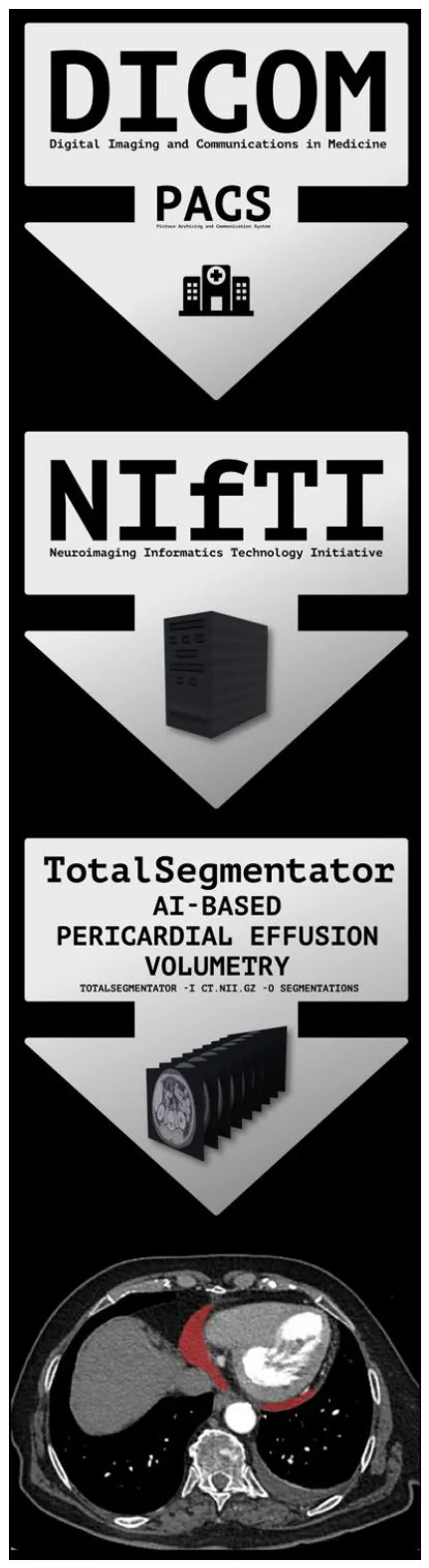
Schematic overview of the AI-based pericardial effusion quantification workflow. Routine electrocardiography-gated pre-procedural CT angiography datasets acquired for TAVI planning were processed using Total Segmentator, a deep learning-based framework for automated anatomical segmentation of CT images. The pericardial space was automatically delineated. AI-generated segmentations were reviewed for quality control by a radiology resident with 3 years of cardiovascular CT experience under supervision of an attending cardiovascular radiologist. Pericardial fluid volume was quantified in milliliters (mL). Datasets with insufficient image quality or inadequate segmentation were excluded. The resulting volumetric measurements were used for all categorical, regression, and spline analyses.

Pericardial effusion volume was extracted from the AI-generated segmentation mask and quantified in milliliters (mL). All segmentations underwent visual quality control by a radiology resident with 3 years of cardiovascular CT experience under the supervision of an attending cardiovascular radiologist. No manual corrections of AI-generated segmentations were performed. Cases with insufficient image quality or inadequate segmentation quality were excluded prior to analysis. The resulting volumetric measurements were used for all subsequent analyses. A schematic overview of the AI-based quantification workflow is provided in Figure 1.

### 2.4. Definition of Pericardial Effusion Categories

For descriptive and survival analyses, patients were categorized according to AI-derived pericardial effusion volume into four groups: no effusion (0 mL), trace effusion (>0 to <5 mL), small effusion (5 to <30 mL), and larger effusion (≥30 mL).

These categories were chosen to represent increasing degrees of pericardial fluid accumulation while maintaining clinically meaningful group sizes In addition to categorical analyses, pericardial effusion volume was evaluated as a continuous variable. Because of its right-skewed distribution, log-transformed pericardial effusion volume was used for regression and spline analyses.

### 2.5. Echocardiographic Assessment

As part of routine clinical evaluation before TAVI, all patients underwent comprehensive transthoracic echocardiography within the pre-procedural assessment period. Examinations were performed using dedicated cardiovascular ultrasound platforms (iE33 and EPIQ 5, Philips Healthcare, Hamburg, Germany) and analyzed by experienced operators specialized in structural heart disease imaging.

Echocardiographic assessment included characterization of aortic stenosis severity, left ventricular systolic function, transvalvular hemodynamics, and right heart performance. Left ventricular ejection fraction (LVEF) was determined using established volumetric techniques, while stroke volume index (SVi) was derived from Doppler measurements of the left ventricular outflow tract and indexed to body surface area.

Right-sided hemodynamics were evaluated by estimating systolic pulmonary artery pressure (sPAP) from tricuspid regurgitation velocity combined with an echocardiographic estimate of right atrial pressure. Right ventricular longitudinal systolic function was assessed by tricuspid annular plane systolic excursion (TAPSE). To provide an integrated measure of right ventricular–pulmonary arterial interaction, the TAPSE/sPAP ratio was calculated and used as a marker of right ventricular–pulmonary arterial coupling.

All measurements were obtained and interpreted according to contemporary recommendations of the European Association of Cardiovascular Imaging and current valvular heart disease guidelines [18,19,20,21].

### 2.6. TAVI Procedure

All procedures were performed via transfemoral access using contemporary self-expanding transcatheter heart valve systems, including CoreValve™ Evolut™ R and CoreValve™ Evolut™ Pro (Medtronic Inc., Minneapolis, MN, USA).

Pre-procedural assessment included transthoracic echocardiography and contrast-enhanced CT angiography for procedural planning, valve sizing, and vascular access evaluation. All interventions were performed according to current guideline recommendations and institutional practice standards by experienced structural heart teams.

### 2.7. Outcome Assessment

The primary endpoint of the study was all-cause mortality following TAVI. Vital status was obtained through review of hospital records, structured follow-up telephone contact with patients or their treating physicians, and verification through official death registries. Follow-up duration was calculated from the date of TAVI to death or last available clinical contact.

### 2.8. Statistical Analysis

Statistical analyses were performed using R (version 4.2.3; R Foundation for Statistical Computing, Vienna, Austria). Continuous variables are presented as mean ± standard deviation (SD) or median with interquartile range (IQR), as appropriate. Categorical variables are reported as frequencies and percentages. Comparisons between pericardial effusion groups were performed using one-way analysis of variance (ANOVA), the Kruskal–Wallis test, or the chi-square test, as appropriate.

Long-term survival was evaluated using Kaplan–Meier analysis, and differences between groups were assessed using the log-rank test. Associations between pericardial effusion volume and all-cause mortality were examined using Cox proportional hazards regression models and are reported as hazard ratios (HRs) with 95% confidence intervals (CIs). Pericardial effusion volume was analyzed both as a continuous variable and according to predefined volumetric categories. Additional analyses were performed using binary thresholds of ≥5 mL, ≥10 mL, and ≥30 mL, as well as the presence of any detectable pericardial effusion (>0 mL).

Multivariable Cox regression analyses were adjusted for clinically relevant covariates selected a priori, including age, sex, atrial fibrillation, New York Heart Association (NYHA) functional class ≥III, systolic pulmonary artery pressure (sPAP), and the TAPSE/sPAP ratio. As a sensitivity analysis, an extended multivariable Cox regression model additionally adjusted for diabetes mellitus, COPD, renal dysfunction (defined as serum creatinine ≥1.5 mg/dL), and history of malignancy.

To investigate potential non-linear associations between pericardial effusion volume and mortality risk, restricted cubic spline analyses with four knots were performed using log-transformed pericardial effusion volume. Overall and non-linear associations were evaluated using Wald statistics.

All statistical tests were two-sided, and a *p*-value <0.05 was considered statistically significant.

## 3. Results

### 3.1. Distribution of AI-Derived Pericardial Effusion Volume

The distribution of AI-derived pericardial effusion volume is summarized in Table 1.

Among the 470 patients included in the study, 194 (41.3%) exhibited no detectable pericardial effusion. Trace effusions (>0 to <5 mL) were identified in 181 patients (38.5%), whereas 71 patients (15.1%) had small effusions ranging from 5 to <30 mL. Larger effusions (≥30 mL) were present in only 24 patients (5.1%).

Overall, detectable pericardial effusion was observed in 276 patients (58.7%). However, the vast majority of effusions were small in volume, with only a minority of patients demonstrating larger fluid accumulations exceeding 30.

### 3.2. Baseline Characteristics According to Pericardial Effusion Volume

Baseline characteristics stratified according to AI-derived pericardial effusion volume are summarized in Table 2.

Demographic characteristics were largely comparable across groups. Age, sex distribution, body mass index, and body surface area did not differ significantly according to pericardial effusion burden. Similarly, the prevalence of diabetes mellitus, chronic obstructive pulmonary disease, prior stroke, and history of malignancy was comparable between groups.

In contrast, several clinically relevant differences were observed. The prevalence of atrial fibrillation increased progressively with higher pericardial effusion volumes, ranging from 30.9% in patients without pericardial effusion to 58.3% in patients with larger effusions (*p* = 0.001). Significant differences were also observed for NYHA functional class ≥III (*p* = 0.024).

Echocardiographic parameters demonstrated a consistent relationship between increasing pericardial effusion burden and markers of impaired right heart function. Patients with larger effusions exhibited higher systolic pulmonary artery pressure (sPAP) values (*p* = 0.004) and lower TAPSE/sPAP ratios (*p* = 0.007). A trend toward lower stroke volume index was observed across increasing pericardial effusion categories, although this did not reach statistical significance (*p* = 0.073). No significant differences were detected for left ventricular ejection fraction or mean transvalvular aortic valve gradient.

### 3.3. Pericardial Effusion Volume and Long-Term Survival

**Figure 2:**
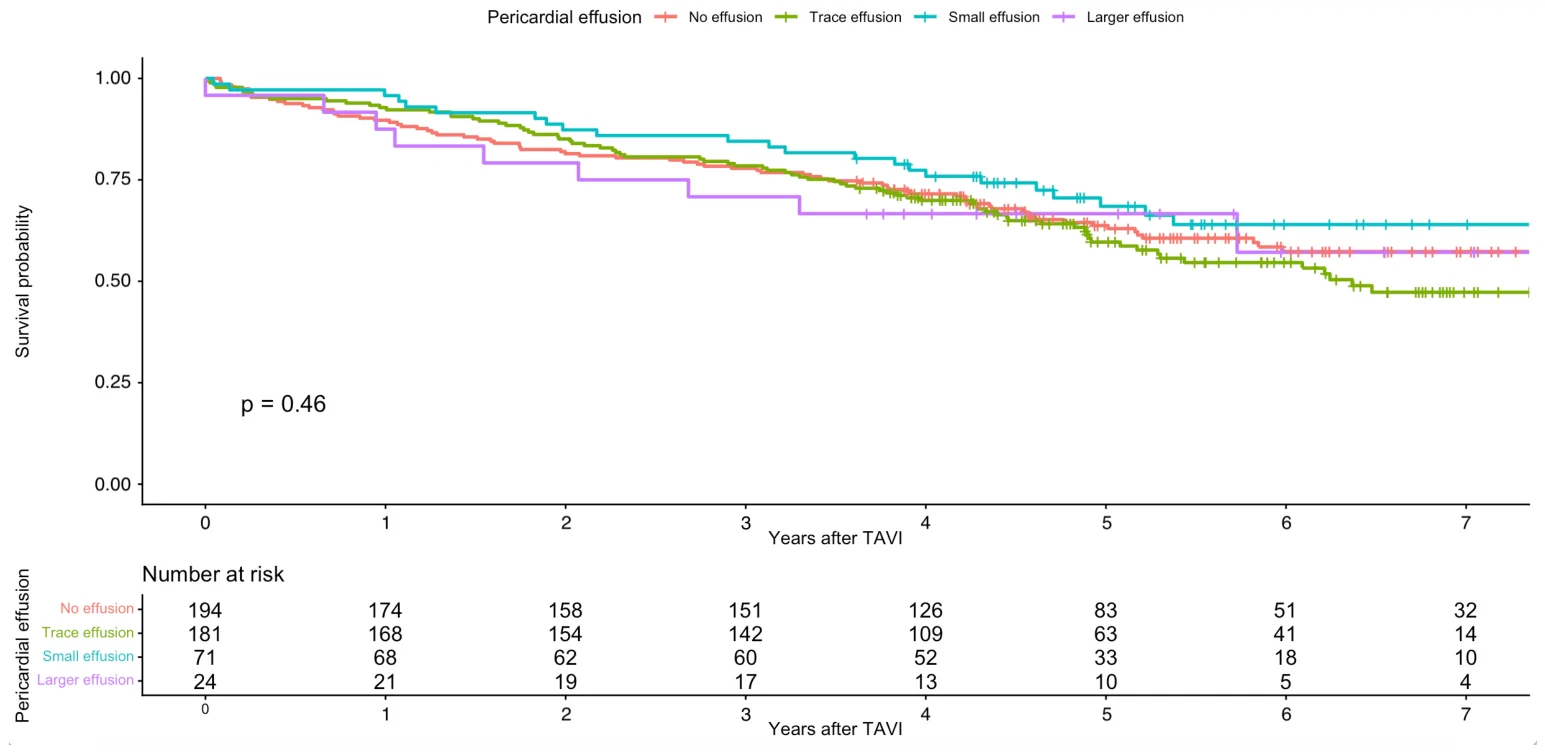
Kaplan–Meier estimates of long-term survival after TAVI stratified by AI-derived pericardial effusion category: no effusion (0 mL), trace effusion (>0 to <5 mL), small effusion (5 to <30 mL), and larger effusion (≥30 mL). The *x*-axis shows time.

Kaplan–Meier survival curves according to pericardial effusion category are presented in Figure 2.

During long-term follow-up, no significant differences in all-cause mortality were observed among patients with no effusion, trace effusion, small effusion, or larger effusion. Survival estimates remained largely comparable throughout follow-up, and the overall log-rank test did not demonstrate a significant association between pericardial effusion category and mortality (log-rank *p* = 0.46).

### 3.4. Cox Regression Analyses

The association between pericardial effusion volume and long-term mortality was further assessed using Cox proportional hazards regression models (Table 3).

When analyzed as a continuous variable, pericardial effusion volume was not associated with mortality (HR 1.00, 95% CI 0.996–1.01; *p* = 0.842). Similar findings were observed after logarithmic transformation of pericardial effusion volume (HR 0.93, 95% CI 0.80–1.08; *p* = 0.340).

Likewise, the presence of any detectable pericardial effusion (>0 mL) was not associated with mortality risk (HR 1.03, 95% CI 0.77–1.38; *p* = 0.821). Additional analyses using predefined volumetric thresholds of ≥5 mL, ≥10 mL, and ≥30 mL similarly demonstrated no significant associations with long-term mortality.

After adjustment for age, sex, atrial fibrillation, NYHA functional class ≥III, sPAP, and TAPSE/sPAP, log-transformed pericardial effusion volume remained unassociated with mortality (adjusted HR 0.91, 95% CI 0.76–1.08; *p* = 0.293). Compared with patients without pericardial effusion, neither trace effusions (HR 1.30, 95% CI 0.90–1.87; *p* = 0.157), small effusions (HR 0.75, 95% CI 0.44–1.30; *p* = 0.308), nor larger effusions (HR 1.23, 95% CI 0.57–2.65; *p* = 0.598) were associated with increased mortality risk.

In an additional sensitivity analysis including diabetes mellitus, COPD, renal dysfunction, and history of malignancy, the association between AI-derived pericardial effusion volume and long-term mortality remained unchanged (HR per 1-SD increase 0.90, 95% CI 0.75–1.08; *p* = 0.247). Likewise, categorical analyses comparing trace, small, and larger pericardial effusions with patients without pericardial effusion yielded similar results.

### 3.5. Restricted Cubic Spline Analysis

**Figure 3:**
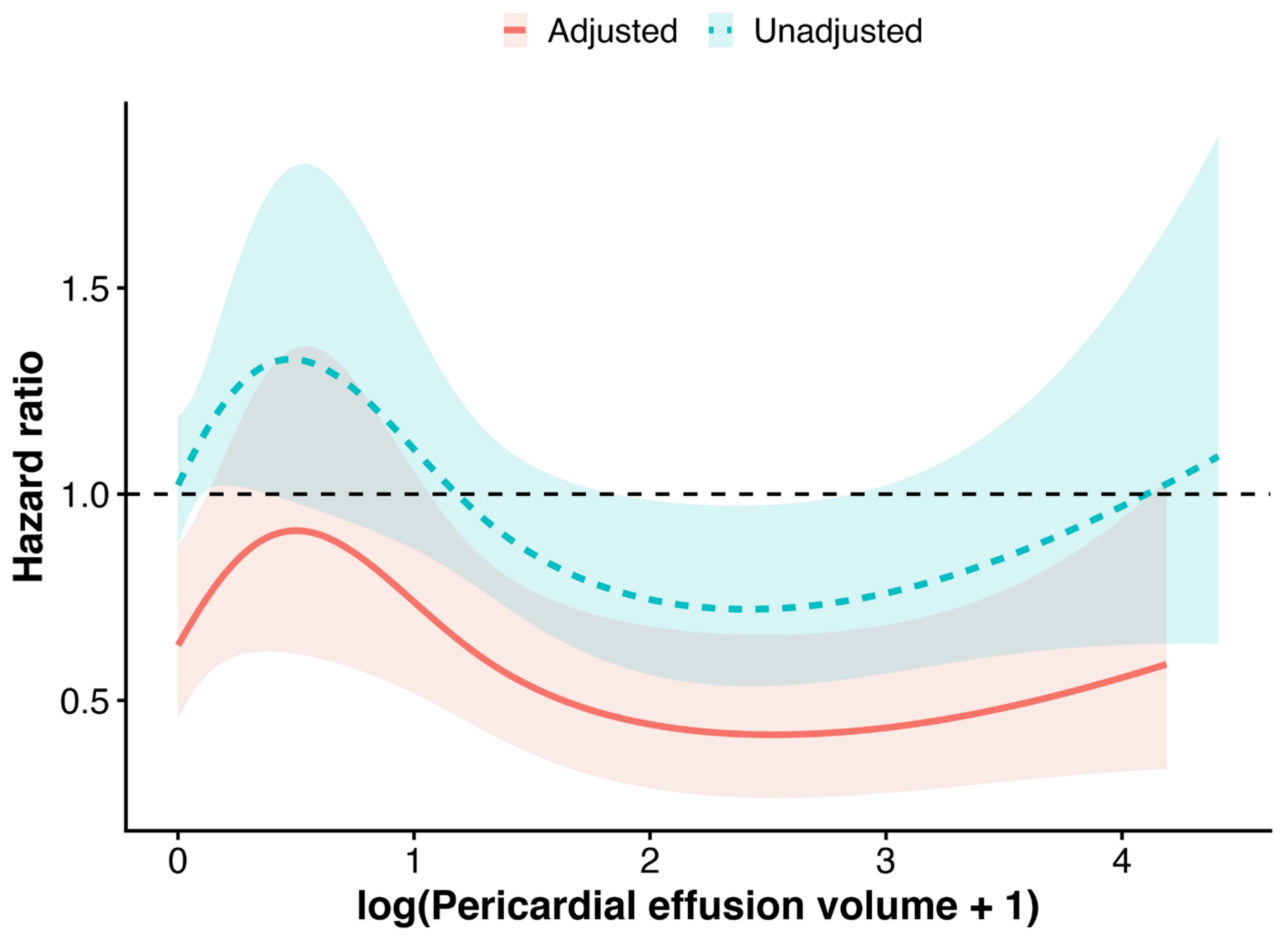
Restricted cubic spline analysis of the association between log-transformed pericardial effusion volume [log(pericardial effusion volume + 1)] and the hazard of long-term all-cause mortality after TAVI. The solid red and dashed turquoise curves represent the adjusted and unadjusted estimates, respectively; shaded areas indicate 95% confidence intervals. The horizontal dashed line denotes the reference hazard ratio of 1.0.

To further explore the possibility of non-linear associations between pericardial effusion volume and long-term mortality, restricted cubic spline analyses were performed using log-transformed pericardial effusion volume (Figure 3, Table 4).

In the unadjusted model, neither the overall association between pericardial effusion volume and mortality (Wald χ^2^ = 5.23, *p* = 0.156) nor the non-linear component (Wald χ^2^ = 4.59, *p* = 0.101) reached statistical significance.

Similar findings were observed after multivariable adjustment. The overall association remained non-significant (Wald χ^2^ = 6.15, *p* = 0.105), and no statistically significant evidence of non-linearity was detected (Wald χ^2^ = 5.38, *p* = 0.068).

Across the observed range of pericardial effusion volumes, no clear increase in mortality risk was identified, supporting the results of the categorical and regression-based analyses.

## 4. Discussion

### 4.1. Main Findings

The present study evaluated the clinical and prognostic significance of AI-derived pericardial effusion volume in patients undergoing TAVI. Pericardial effusions were frequently observed on routine pre-procedural CT imaging and were associated with distinct differences in clinical and echocardiographic characteristics, particularly parameters reflecting right-heart dysfunction and hemodynamic burden. Despite these associations, neither categorical nor continuous measures of pericardial effusion volume were related to long-term mortality. Moreover, comprehensive analyses using multivariable Cox regression and restricted cubic spline modelling consistently failed to demonstrate an independent prognostic effect across the entire spectrum of pericardial effusion volumes.

### 4.2. Pericardial Effusion in Aortic Stenosis: A Marker of Hemodynamic Burden

The mechanisms underlying pericardial effusion formation in patients with severe aortic stenosis are likely multifactorial. While pericardial effusions are traditionally associated with inflammatory [22], infectious [23] or malignant [24] disorders, fluid accumulation in elderly patients undergoing TAVI is more likely to reflect chronic hemodynamic alterations than intrinsic pericardial disease. Progressive pressure overload caused by severe aortic stenosis initiates a cascade of structural and functional adaptations, including left ventricular hypertrophy, impaired diastolic filling, left atrial remodeling, pulmonary hypertension, and eventually right-heart dysfunction [14,25]. As filling pressures rise and systemic venous congestion develops, transudation of fluid into serosal compartments may occur, resulting in pleural and pericardial effusions [26].

Our findings support this pathophysiological concept. Patients with increasing pericardial effusion volumes exhibited a higher prevalence of atrial fibrillation, elevated systolic pulmonary artery pressure, and lower TAPSE/sPAP ratios, indicating more advanced impairment of right ventricular–pulmonary arterial coupling. In contrast, left ventricular ejection fraction and transvalvular gradients remained largely comparable across effusion categories. Collectively, these observations suggest that pericardial effusion volume primarily reflects the hemodynamic consequences of advanced cardiovascular remodeling and congestion rather than the severity of valvular obstruction itself. From this perspective, pericardial effusion may be viewed as part of a broader congestion phenotype frequently encountered in elderly patients undergoing TAVI.

### 4.3. Pericardial Effusion and Long-Term Mortality After TAVI

Despite its association with markers of hemodynamic burden, AI-derived pericardial effusion volume was not associated with long-term mortality in our cohort. This observation remained remarkably consistent across categorical analyses, continuous modeling approaches, multivariable adjustment, additional sensitivity analyses with extended adjustment for major extracardiac comorbidities, and restricted cubic spline analyses, suggesting that the absence of a prognostic signal is unlikely to be explained by methodological considerations alone.

At first glance, the lack of an independent association with mortality may appear unexpected. However, pericardial effusion is likely better understood as a marker of underlying cardiovascular stress than as a direct mediator of adverse outcomes [5,26]. Similar to other manifestations of congestion, the accumulation of pericardial fluid may reflect the cumulative effects of elevated filling pressures, pulmonary hypertension, and progressive cardiac remodeling [27]. In this regard, pericardial effusion may represent a consequence of advanced disease rather than a determinant of subsequent mortality.

This interpretation is further supported by the distribution of effusion volumes observed in the present study. Although detectable pericardial fluid was identified in nearly 60% of patients, larger effusions were uncommon and the majority of fluid collections were small in volume. Such findings may be sufficient to indicate underlying hemodynamic burden while remaining clinically silent from a prognostic perspective. The presence of pericardial fluid therefore appears to provide information about the patient’s physiological state rather than about future survival.

The clinical context may also be important when interpreting these findings. Previous studies conducted in patients with acute [28] or chronic heart failure [29], advanced pulmonary hypertension [27], or other forms of severe cardiovascular disease have linked pericardial effusions to adverse outcomes. In contrast, the present cohort consisted of patients undergoing comprehensive evaluation and treatment of severe aortic stenosis. Following successful valve replacement, the hemodynamic abnormalities contributing to pericardial fluid accumulation may partially improve, potentially attenuating the long-term prognostic implications of a pre-existing effusion.

### 4.4. Artificial Intelligence and Comprehensive Phenotyping Beyond the Aortic Valve

Beyond the clinical implications of pericardial effusion itself, the present study highlights the growing potential of artificial intelligence to extract clinically meaningful information from routinely acquired cardiovascular imaging. Contemporary TAVI planning CT scans contain substantially more information than is currently incorporated into clinical decision-making. Traditionally, image analysis has focused primarily on valve sizing, annular anatomy, and vascular access planning [30], whereas many additional anatomical features are either assessed qualitatively or overlooked entirely.

Recent advances in AI-based image analysis have begun to transform this cardiovascular paradigm by enabling automated, reproducible, and scalable quantification of structures that were previously difficult to evaluate systematically [31]. In the present study, AI-derived volumetric assessment of pericardial effusion allowed objective characterization of a frequently encountered but poorly understood imaging finding across an entire TAVI population. Although pericardial effusion volume was not associated with long-term mortality, its relationship with markers of hemodynamic burden demonstrates how AI can uncover clinically relevant phenotypes that extend beyond traditional measures of valvular disease severity.

These findings support an evolving view of severe aortic stenosis as a complex systemic condition rather than an isolated valvular disorder [32]. The clinical trajectory of patients undergoing TAVI is influenced not only by the severity of valve obstruction but also by cardiac remodeling, pulmonary vascular disease, congestion, frailty, and biological aging [13,25]. Artificial intelligence offers a unique opportunity to capture these dimensions from routine imaging datasets and may contribute to a more comprehensive characterization of patient vulnerability in the future. Rather than replacing established clinical and echocardiographic parameters, AI-derived imaging biomarkers may complement existing risk assessment strategies by revealing aspects of disease biology that are not readily apparent during conventional image interpretation.

## 5. Limitations

This study should be interpreted in light of several limitations. The retrospective single-center design may limit the generalizability of the findings and does not eliminate the possibility of residual confounding despite multivariable adjustment. Nevertheless, the analysis was based on a large, well-characterized real-world TAVI cohort with comprehensive long-term follow-up. Pericardial effusion volume was assessed at a single pre-procedural time point, precluding evaluation of temporal changes following valve replacement. Future studies incorporating serial imaging may help clarify whether dynamic changes in pericardial effusion burden provide additional clinical or prognostic information. The relatively small number of patients with larger pericardial effusions should also be considered when interpreting the results. However, this distribution reflects contemporary TAVI populations, in which substantial pericardial fluid accumulation is uncommon and most effusions are small in volume. Finally, invasive hemodynamic measurements were not routinely available. Consequently, the relationship between pericardial effusion volume and congestion was inferred from established clinical and echocardiographic markers rather than direct hemodynamic assessment. Although this limits mechanistic interpretation, the consistent associations observed with atrial fibrillation, pulmonary pressures, and right ventricular–pulmonary arterial coupling support the biological plausibility of the findings.

## 6. Conclusions

In patients undergoing TAVI, AI-derived pericardial effusion volume is a common imaging finding and is associated with markers of hemodynamic burden, including pulmonary hypertension, atrial fibrillation, and impaired right ventricular–pulmonary arterial coupling. Despite these associations, pericardial effusion volume was not independently associated with long-term mortality. These findings suggest that pericardial effusion may be interpreted as a marker of cardiovascular congestion and advanced cardiac remodeling rather than a prognostically relevant phenotype. AI-based volumetric assessment of routine CT imaging may provide novel opportunities for comprehensive phenotyping of patients with severe aortic stenosis beyond the aortic valve itself.

## Figures and Tables

**Table 1:** Distribution of AI-Derived Pericardial Effusion Volume.

Group	Definition	*n* (%)
No effusion	0 mL	194 (41.3)
Trace effusion	>0 to <5 mL	181 (38.5)
Small effusion	5 to <30 mL	71 (15.1)
Larger effusion	≥30 mL	24 (5.1)

Distribution of AI-derived pericardial effusion volume across the study population (*n* = 470). Patients are grouped by volumetric category—no effusion (0 mL), trace effusion (>0 to <5 mL), small effusion (5 to <30 mL), and larger effusion (≥30 mL)—with the number and percentage of patients in each group. Detectable pericardial effusion was present in 276 patients (58.7%), but larger effusions were uncommon (5.1%), with most fluid collections being small in volume.

**Table 2:** Baseline Characteristics According to Pericardial Effusion Group.

Variable	Overall	No Effusion	Trace Effusion	Small Effusion	Larger Effusion	*p*-Value
Demographics						
Age, years	82 [79–86]	83 [79–86]	82 [79–85]	82 [79–86]	82 [78.8–85]	0.979
Male sex, *n* (%)	239 (50.9)	106 (54.6)	89 (49.2)	36 (50.7)	8 (33.3)	0.234
BMI, kg/m^2^	25.2 [22.7–28.2]	25.3 [23.1–28.4]	25.3 [22.7–27.8]	24.7 [22.2–29.1]	24.4 [22.5–26.6]	0.403
BSA, m^2^	1.82 [1.68–1.95]	1.83 [1.70–1.96]	1.80 [1.65–1.94]	1.83 [1.69–1.97]	1.81 [1.68–1.88]	0.529
Clinical Characteristics						
NYHA ≥ III, *n* (%)	153 (38.1)	74 (45.1)	51 (32.5)	24 (40.7)	4 (18.2)	0.024
Diabetes mellitus, *n* (%)	122 (26.0)	57 (29.4)	43 (23.8)	16 (22.5)	6 (25.0)	0.555
Atrial fibrillation, *n* (%)	175 (37.2)	60 (30.9)	63 (34.8)	38 (53.5)	14 (58.3)	0.001
COPD, *n* (%)	52 (11.1)	16 (8.2)	27 (14.9)	7 (9.9)	2 (8.3)	0.205
Prior stroke, *n* (%)	39 (8.3)	17 (8.8)	16 (8.8)	4 (5.6)	2 (8.3)	0.853
History of malignancy, *n* (%)	77 (16.4)	30 (15.5)	26 (14.4)	15 (21.1)	6 (25.0)	0.373
Laboratory Parameters						
Hemoglobin, g/dL	12.9 [11.8–14.0]	12.9 [11.9–14.0]	12.9 [12.1–14.0]	12.4 [11.4–13.6]	12.9 [11.3–14.4]	0.169
Creatinine, mg/dL	1.00 [0.83–1.30]	1.04 [0.82–1.24]	0.96 [0.82–1.27]	1.15 [0.83–1.40]	1.00 [0.86–1.33]	0.295
Echocardiographic Parameters						
LVEF, %	55 [50–60]	55 [50–60]	55 [50–60]	55 [50–60]	55 [47.5–60]	0.997
Mean AV gradient, mmHg	44.3 [39–50]	45 [38.2–50]	45 [39–51]	44 [40–50.5]	44.5 [40.8–54]	0.852
sPAP, mmHg	37 [27–48.5]	35 [26–45]	36 [26–48]	45 [32.3–56.3]	40.5 [28.8–51.5]	0.004
TAPSE, mm	21.9 [19–25]	22 [19–25]	22 [19–25]	20 [18–24]	19.5 [17.8–23.3]	0.087
TAPSE/sPAP	0.60 [0.42–0.87]	0.61 [0.47–0.91]	0.62 [0.42–0.91]	0.47 [0.34–0.69]	0.48 [0.35–0.75]	0.007
Stroke volume index, mL/m^2^	43.7 [35.3–55.7]	45.8 [37.8–55.8]	43.6 [33.0–55.9]	41.1 [34.3–55.5]	40.5 [32.2–46.2]	0.073

Baseline demographic, clinical, laboratory, and echocardiographic characteristics of the study population, overall and stratified by AI-derived pericardial effusion group. Continuous variables are presented as median [interquartile range] and categorical variables as *n* (%). Between-group comparisons were performed using ANOVA, the Kruskal–Wallis test, or the chi-square test, as appropriate; *p*-values refer to the comparison across all four effusion groups. Increasing effusion volume was associated with a higher prevalence of atrial fibrillation (*p* = 0.001), higher NYHA class ≥III (*p* = 0.024), higher sPAP (*p* = 0.004), and lower TAPSE/sPAP ratio (*p* = 0.007), whereas age, sex, BMI, BSA, LVEF, and mean transvalvular aortic gradient did not differ significantly. BMI, body mass index; BSA, body surface area; NYHA, New York Heart Association; COPD, chronic obstructive pulmonary disease; LVEF, left ventricular ejection fraction; AV, aortic valve; sPAP, systolic pulmonary artery pressure; TAPSE, tricuspid annular plane systolic excursion.

**Table 3:** Cox Regression Analyses of Pericardial Effusion and Long-Term Mortality After TAVI.

Variable	HR (95% CI)	*p*-Value	Model
Pericardial effusion volume (per 1 mL)	1.00 (0.996–1.01)	0.842	Unadjusted
Log-transformed PE volume (per 1 SD)	0.93 (0.80–1.08)	0.340	Unadjusted
Any pericardial effusion (>0 mL)	1.03 (0.77–1.38)	0.821	Unadjusted
Pericardial effusion ≥5 mL	0.80 (0.55–1.16)	0.234	Unadjusted
Pericardial effusion ≥10 mL	0.94 (0.61–1.46)	0.783	Unadjusted
Pericardial effusion ≥30 mL	1.03 (0.53–2.02)	0.926	Unadjusted
Log-transformed PE volume (per 1 SD)	0.91 (0.76–1.08)	0.293	Adjusted *
Trace effusion vs. no effusion	1.30 (0.90–1.87)	0.157	Adjusted *
Small effusion vs. no effusion	0.75 (0.44–1.30)	0.308	Adjusted *
Larger effusion vs. no effusion	1.23 (0.57–2.65)	0.598	Adjusted *
Log-transformed PE volume (per 1 SD)	0.90 (0.75–1.08)	0.247	Adjusted **
Trace effusion vs. no effusion	1.28 (0.89–1.86)	0.183	Adjusted **
Small effusion vs. no effusion	0.74 (0.43–1.29)	0.288	Adjusted **
Larger effusion vs. no effusion	1.21 (0.55–2.64)	0.635	Adjusted **

* Adjusted for age, sex, atrial fibrillation, NYHA class ≥III, sPAP, and TAPSE/sPAP. ** Adjusted for age, sex, atrial fibrillation, NYHA class ≥III, sPAP, TAPSE/sPAP, diabetes, COPD, renal dysfunction (creatinine ≥ 1.5 mg/dL) and history of malignancy. Cox proportional hazards regression analyses of the association between pericardial effusion and long-term all-cause mortality after TAVI. Hazard ratios (HR) are reported with 95% confidence intervals (CI). Pericardial effusion was modeled as a continuous variable (per 1 mL and per 1 SD of log-transformed volume), as any detectable effusion (>0 mL), using binary thresholds (≥5, ≥10, and ≥30 mL), and as categorical groups versus no effusion. Adjusted models account for age, sex, atrial fibrillation, NYHA class ≥III, sPAP, and TAPSE/sPAP. No measure of pericardial effusion was significantly associated with mortality in unadjusted or adjusted analyses. PE, pericardial effusion; SD, standard deviation.

**Table 4:** Restricted cubic spline analyses of log-transformed pericardial effusion volume and long-term mortality.

Model	Overall Association (Wald χ^2^)	df	*p*-Overall	Non-Linearity (Wald χ^2^)	df	*p*-Nonlinearity
Unadjusted model	5.23	3	0.156	4.59	2	0.101
Adjusted model *	6.15	3	0.105	5.38	2	0.068

* Adjusted for age, sex, atrial fibrillation, NYHA class ≥III, sPAP, and TAPSE/sPAP. Restricted cubic spline analyses (four knots) of log-transformed pericardial effusion volume and long-term all-cause mortality, for the unadjusted and adjusted models. The table reports the Wald χ^2^ statistic, degrees of freedom (df), and *p*-value for both the overall association and the non-linear component. Neither the overall association nor the non-linear term reached statistical significance in either model, indicating no evidence of a linear or non-linear relationship between pericardial effusion volume and mortality.

## Data Availability

The data presented in this study are available on reasonable request from the corresponding author. The data are not publicly available due to privacy and institutional restrictions.
